# Stigma, Bullying, and Resilience: Psychosocial Outcomes in Marginalized Adolescents With Visible Dermatologic Conditions

**DOI:** 10.7759/cureus.86171

**Published:** 2025-06-16

**Authors:** Andres D Parga, Taryn M Liska

**Affiliations:** 1 Medicine, Hospital Corporation of America (HCA) Florida Oak Hill Hospital, Brooksville, USA; 2 Intensive Care Unit, Banner Health University Medical Center, Phoenix, USA

**Keywords:** acne, alopecia areata, atopic dermatitis, dermatology, hidradenitis suppurativa, pediatric dermatology, psoriasis, psychodermatology, skin of color, vitiligo

## Abstract

Visible skin diseases such as acne, eczema, vitiligo, and keloids exert a disproportionate psychosocial burden on marginalized adolescents, particularly among Indigenous, Latinx, and migrant youth. This review illuminates how the cycle of stigma, bullying (both in-person and online), and discrimination is amplified by intersecting cultural, racial, and socioeconomic factors, leading to elevated rates of depression, anxiety, self-harm, and suicide risk. However, despite the gravity of these challenges, psychosocial distress remains routinely underdiagnosed and undertreated in these populations, with significant barriers to mental health care and a lack of culturally competent interventions. Drawing from a broad and interdisciplinary evidence base, we explore not only the epidemiology and risk factors unique to minoritized youth but also the powerful influence of cultural identity, family support, traditional practices, and peer mentorship in shaping resilience. Our review uniquely highlights integrative and creative therapies as promising adjuncts to conventional care. We emphasize the centrality of internalized stigma as a therapeutic target and urge a shift toward trauma-informed care. The findings presented call for coordinated action among dermatologists, pediatricians, psychiatrists, psychologists, educators, and family members as key stakeholders in supporting the psychosocial needs of adolescents with visible skin disease. We recommend systematic psychosocial screening, routine use of validated quality-of-life and stigma measures, family- and community-centered support, and the integration of mental health resources into dermatologic care. Ultimately, by embracing culturally sensitive, multi-level strategies and addressing persistent policy and research gaps, we can not only mitigate the harms of visible skin disease but also transform vulnerability into resilience and agency for the most underserved youth.

## Introduction and background

Psychodermatology is a growing interdisciplinary field examining the complex interplay between the mind and skin, rooted in the shared embryological origin of the nervous system and cutaneous tissues [1]. In youth, this relationship is particularly salient, as visible skin diseases frequently emerge during key developmental periods, intersecting with identity formation, peer relationships, and evolving self-esteem [2,3]. Approximately 30% of chronic skin conditions are influenced by psychiatric disturbances, and the prevalence of psychosocial comorbidities, including depression, anxiety, and suicidality, is especially high among adolescents with visible dermatologic disease [1,4]. The psychosocial burden of visible skin disorders is not evenly distributed across all individuals. Marginalized youth, particularly those from Indigenous, Latinx, and migrant communities, face heightened risk due to the compounding effects of structural discrimination, colorism, racism, and socioeconomic adversity [5,6]. Colorism, or the social stratification based on skin color, is a powerful determinant of health, operating both across and within racial and ethnic groups. Darker-skinned youth, especially females, are disproportionately exposed to discrimination, chronic stress, and stigma, leading to higher rates of depressive symptoms, obesity, and poor self-reported health [5]. Bullying and xenophobia further amplify risk, targeting stable and uncontrollable aspects of identity such as skin color, culture, and citizenship, with significant consequences for mental health, academic performance, and social functioning [6,7]. Despite the profound impact of visible skin disease on psychosocial outcomes, psychodermatologic care remains under-emphasized in dermatology practice, especially for marginalized youth. Most dermatologists and psychiatrists receive little training in psychodermatology, and there are significant gaps in culturally competent care for Indigenous, Latinx, and other underrepresented groups [8]. This lack of focus has perpetuated healthcare disparities and hindered the implementation of trauma-informed, interdisciplinary interventions that are urgently needed for these vulnerable populations. In summary, integrating psychodermatologic principles into dermatology care is essential for addressing the unique and disproportionate burdens faced by marginalized youth with visible skin disease. This review aims to synthesize current evidence on the epidemiology, psychosocial outcomes, and intervention strategies relevant to these high-risk groups, highlighting critical gaps and future directions for research and practice.

## Review

Materials and methods

This narrative review synthesizes published evidence on the psychosocial impacts of visible skin diseases among marginalized adolescents, with a focus on stigma, bullying, and resilience. To capture the evolution of knowledge and emerging trends, we conducted a comprehensive search of English-language literature published in the past 20 years. Databases searched included PubMed, MEDLINE, Embase, PsycINFO, and Google Scholar. Search terms incorporated combinations of “visible skin disease”, “acne”, “eczema”, “vitiligo”, “psoriasis”, “keloids”, “stigma”, “bullying”, “colorism”, “psychosocial”, “resilience”, “minority youth”, “Indigenous”, “Latinx”, “migrant”, “school-based interventions”, and “mental health”. We included peer-reviewed original research (quantitative and qualitative), systematic reviews, meta-analyses, case series, consensus statements, and interdisciplinary commentaries addressing adolescents (ages 10-24) with visible dermatologic conditions and psychosocial outcomes. Studies focusing on epidemiology, mental health, stigma, bullying, discrimination, intersectionality, coping, and interventions in marginalized populations were prioritized. Relevant articles were also identified through citation chaining of included studies. Both global and region-specific studies were included to capture cultural variability, with a special focus on research in North America, Latin America, Europe, and Australia. Exclusion criteria included non-English publications, animal studies, and articles that lacked a focus on psychosocial or marginalized youth outcomes.

All references were reviewed independently by both authors for relevance and quality. Discrepancies regarding study inclusion were resolved by consensus. The final synthesis prioritized studies that provided prevalence data, validated measures (e.g., the Children’s Dermatology Life Quality Index, PROMIS), and intervention outcomes. Hyperlinks to referenced studies are provided in the main text for reader access. Given the narrative scope of this review, the methodology and detailed findings of each study are not described at length to maintain readability and conciseness.

Epidemiology and burden

Visible skin diseases, including acne, atopic dermatitis (eczema), vitiligo, psoriasis, and keloids, are highly prevalent among adolescents globally, with disproportionately higher rates and greater psychosocial impact among minoritized populations such as Indigenous, Latinx, Black, and migrant youth (Table 1) [2,3,8,9]. Acne vulgaris is one of the most common skin disorders in adolescence, affecting up to 85% of teens in the general population and up to 89% in some high-prevalence regions [8,10]. Prevalence rates remain similar across ethnic groups; however, the clinical presentation, severity, and risk of psychosocial sequelae may be accentuated in minoritized populations due to intersecting factors, including genetic predisposition, environmental exposures, and systemic inequities (Table 2) [2,5]. Notably, keloids, an example of abnormal wound healing, are far more common in individuals with skin of color, including African American, Latinx, and Asian adolescents, reflecting both genetic susceptibility (e.g., transforming growth factor beta (TGF-β) pathway polymorphisms) and environmental triggers such as acne, piercings, or injury [9]. Atopic dermatitis (eczema) affects approximately 13% of children and 7% of adults in the United States, with even higher rates in certain urban and low-income populations [4]. Minority youth often face greater disease severity, chronicity, and associated psychosocial burden, driven by both intrinsic (e.g., genetic) and extrinsic (e.g., pollution, limited access to care) risk factors [3,11]. Vitiligo, although less prevalent, poses a unique burden due to its high visibility and stigmatization. Prevalence estimates range from 0.5% to 2% in children worldwide, with higher psychosocial impact among darker-skinned youth due to cultural perceptions, colorism, and increased risk of discrimination [3,5,12]. Psoriasis and other chronic inflammatory skin diseases also show increased risk for mental health comorbidities in minoritized groups, particularly in the context of limited access to specialty care and high disease visibility [11,12].

**Table 1:** Epidemiology and psychosocial impact of visible skin disease in marginalized youth SGM: sexual and gender minority, US: United States, AD: atopic dermatitis, QoL: quality of life, RR: relative risk, NSSIB: non-suicidal self-injury behavior, CES-D: Center for Epidemiologic Studies Depression Scale, ↑: increase, ↓: decrease, OR: odds ratio, y/o: years old, “varied”: variability across studies, “parent-report”: data reported by parents/caregivers

Condition/Population	Key Prevalence Data	Major Psychosocial Outcomes	Stigma/Bullying Data	Suicidality (%)	Key References
Acne (global adolescents)	Prevalence: 28.3% (16–24 y/o)	Fatigue (50%), sleep issues (41%)	31% report exclusion; “selfie phobia”; major self-image impact	Up to 25% of those with severe acne report suicidal ideation	Pierre Fabre Laboratories presents the first global study on the “epidemiology of acne”, 2024 [13]; Kelly et al., 2021 [14]; Halvorsen et al., 2011 [15]
Acne (SGM youth, US)	6.2% sexual minorities vs. 3.9% heterosexual	↑ depression, anxiety, poor QoL	↑ stigma, intersectional minority stress	Suicidal ideation: 35.4% (SGM+acne)	Gao et al., 2017 [16]; Ragmanauskaite et al., 2020 [17]
AD	~13% children, ~7% adults (US)	Poor self-esteem, academic problems	20% bullying (US), 18–60% (varied)	Suicidal ideation OR 1.44; attempts OR 1.36	Sandhu et al., 2019 [4]; Kelly et al., 2021 [14]; Magin, 2013 [18]
Psoriasis (pediatric)	7.4%	↓ QoL, intimacy, body image	44% bullied (US), 27% rude remarks	↑ depression/anxiety; RR suicide ~1.3	Kelly et al., 2021 [14]; Picardi et al., 2013 [19]
Chronic pediatric skin disorders	1,671 children at 32 centers	73% experience stigma	29% bullied (parent-report)	14.3% moderate depression	Paller et al., 2024 [3]
NSSIB	~1 in 6 lifetime prevalence	Shame, social withdrawal, anxiety	Often concealed, not reported	Suicide rate 37x higher within 12 months	Paller et al., 2024 [3]
Racist bullying (multiethnic youth)	30–40% (systematic review, global)	Depression, anxiety, ↓ academic engagement	More harmful than general bullying	Not always reported	Sapouna et al., 2023 [6]
Darker-skinned (all races/ethnicities)	Up to 14% fair/poor health (US)	Higher depressive symptoms (CES-D)	↑ discrimination, stress, colorism	Not directly measured	Perreira et al., 2019 [5]

**Table 2:** Unique risk factors in minoritized adolescents with visible skin disease TGF-β: transforming growth factor beta

Risk Factor	Description	Key References
Genetic susceptibility	Certain alleles (e.g., filaggrin mutations in eczema, TGF-β pathway variants in keloids) are more prevalent in populations with skin of color, increasing disease risk and severity.	Miner et al., 2025 [9]
Environmental exposures	Urban crowding, pollution, harsh climates, and occupational exposures (e.g., farmwork, irritants) disproportionately affect marginalized and migrant youth, increasing the prevalence and chronicity of inflammatory and pigmentary disorders.	Paller et al., 2024 [3]; Perreira et al., 2019 [5]
Socioeconomic barriers	Poverty, underinsurance, language barriers, and reduced access to dermatologic care worsen outcomes and delay diagnosis/treatment, especially in Indigenous, Latinx, and immigrant communities.	Paller et al., 2024 [3]; Sapouna et al., 2023 [6]
Discrimination and colorism	Social stratification based on skin color and race increases exposure to stigma, bullying, and chronic stress, compounding both physical and mental health burdens.	Perreira et al., 2019 [5]; Sapouna et al., 2023 [6]

The intersection of these risk factors results in a disproportionate disease burden for marginalized youth, manifesting as higher rates of visible scarring, severe disease, impaired quality of life, and increased risk of psychosocial comorbidities [3,12].

Cycle of stigma: bullying, isolation, and self-image

**Figure 1:**
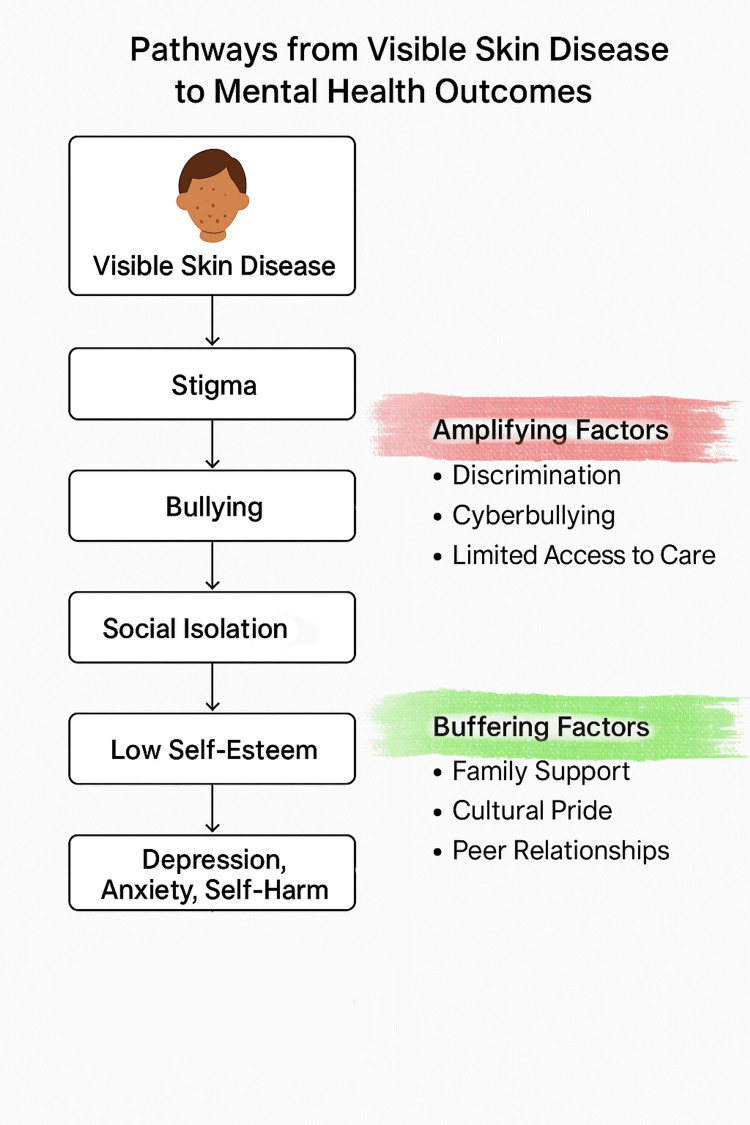
Pathways from visible skin disease to adverse mental health outcomes in marginalized youth Conceptual framework illustrating how visible skin disease can lead to negative mental health outcomes through stigma, bullying, social isolation, and low self-esteem. Amplifying factors (e.g., discrimination, cyberbullying, limited access to care) may worsen these pathways, while buffering factors (e.g., family support, cultural pride, and peer relationships) can provide resilience and mitigate risk. This figure is the authors’ original creation.

Adolescents with visible skin diseases experience a unique and pervasive cycle of stigma characterized by bullying, social isolation, and disrupted self-image processes that are amplified among marginalized youth (Figure 1) [3,6,7].

Bullying and Skin Disease

Both in-person and cyberbullying are disproportionately experienced by youth with visible dermatologic conditions. Meta-analyses estimate that 10-35% of adolescents are recurrently bullied, with those who have visible differences (such as acne, eczema, or keloids) at higher risk due to negative social perceptions and stereotypes [7,20]. Over 60% of teens believe their peers with acne are likely to be bullied, and affected youth report higher rates of embarrassment, lower self-confidence, and difficulties forming friendships and romantic relationships [2,20]. Bullying linked to skin disease is not only frequent but also deeply harmful. Victims are at a significantly increased risk for depression, anxiety, suicidal ideation, loneliness, poor life satisfaction, and even somatic symptoms such as headaches or stomach pain [3,7]. These risks are even higher for youth who face intersectional discrimination based on race, skin color, or cultural markers, such as visible religious dress or ethnic features [5,6].

Cultural and Community Influences

The perception of skin disease and its associated stigma is deeply shaped by cultural context. In some communities, visible differences (like keloids or vitiligo) are sources of shame and social exclusion, while in others, they may be integrated into traditional practices or even valued [9]. However, for many minoritized youth, especially those in Western or assimilative school settings, skin disease often becomes a focal point for negative stereotyping and social exclusion. Internalized stigma, the absorption of society’s negative attitudes, exacerbates self-blame, shame, and social withdrawal [3,12].

Intersections of Skin of Color, Identity, and Discrimination

For youth of color, visible skin conditions intersect with the broader societal dynamics of colorism, racism, and cultural marginalization (Table 3). Darker skin tones are associated with greater exposure to discrimination and chronic stress, compounding the psychosocial impact of dermatologic disease, particularly for girls and gender-diverse youth [5,6,17]. Racialized or racist bullying is especially damaging, targeting immutable aspects of identity and amplifying risks for mental health problems, poor academic performance, and social isolation [6]. Sexual and gender minority adolescents, who already face heightened stigma and bullying, are even more vulnerable to the psychosocial impacts of visible skin disease, reporting greater rates of depression and suicidality [17,21].

**Table 3:** Direct pathways from stigma/bullying to mental health and social outcomes QoL: quality of life, ↑: increase, ↓: decrease, AD: atopic dermatitis, NSSIB: non-suicidal self-injury behavior

Pathway	Empirical Evidence	Main Outcomes	Key Studies
Visible skin disease → social stigma	73% youth report stigma; >50% peer-judged as less desirable	↓ QoL, ↑ depression/anxiety, social exclusion	Paller et al., 2024 [3]; Ritvo et al., 2011 [20]
Social/internalized stigma → mental health	Internalized stigma is the strongest mediator	Depression, anxiety, intimacy/relationship problems	Stuhlmann, 2025 [12]
Bullying/teasing → suicidal ideation	Up to 25% severe acne, 18–20% AD, >20% bullied psoriasis	↑ suicide attempts/ideation, avoidance	Sandhu et al., 2019 [4]; Kelly et al., 2021 [14]
Racist bullying/cultural discrimination → mental health	Systematic review	Depression, distress, withdrawal, ↓ school achievement	Perreira et al., 2019 [5]; Sapouna et al., 2023 [6]
Non-suicidal self-injury → suicide risk	1 in 6 engage in NSSIB; suicide rate 37x higher in 1 year	Death, major morbidity	Paller et al., 2024 [3]

Impact on School Performance and Socialization

The psychological sequelae of bullying, discrimination, and internalized shame are profound: stigmatized youth with skin disease show lower academic engagement, more frequent school absences, reduced participation in extracurricular activities, and impaired peer relationships [2,3,7]. Nearly a third of parents in a recent multi-center study reported that their child had been bullied for their skin disease, an experience strongly correlated with depression, anxiety, and reduced quality of life [3]. Social isolation and avoidance behaviors, such as missing school, skipping sports, or refusing to be photographed, are common coping strategies that further entrench feelings of difference and unworthiness [2,8,20]. For many minoritized adolescents, the cycle of visible difference, bullying, internalized shame, and withdrawal becomes self-perpetuating, undermining both educational achievement and healthy identity development [3,12].

Mental health outcomes

The psychosocial burden of visible skin disease in marginalized adolescents is reflected in disproportionately high rates of depression, anxiety, self-harm, and suicidality, as consistently reported across global studies. These mental health challenges are not only statistically elevated in this population but also have profound implications for overall well-being, school engagement, and social functioning. This section synthesizes evidence on the prevalence, severity, and unique risk factors for psychiatric comorbidities in youth affected by conditions such as acne, atopic dermatitis, vitiligo, psoriasis, and keloids, drawing on recent epidemiologic and clinical research.

Depression, Anxiety, and Suicide Risk

Adolescents with visible skin diseases face substantially increased rates of depression, anxiety, self-harm, and suicidality compared to their healthy peers (Table 4) [2,4,19]. Meta-analyses and population studies have demonstrated that these risks are not only statistically significant but also clinically meaningful: for example, youth with atopic dermatitis are 44% more likely to experience suicidal ideation and 36% more likely to attempt suicide than those without [4]. Nearly one in four adolescents with severe acne reports suicidal thoughts, with girls at especially high risk [17,19]. The risk of self-harm is especially pronounced in this population, as dermatologists are often the first to see signs of non-suicidal self-injury behavior (NSSIB) such as cutting or excoriation. Adolescence is the most common age of onset for NSSIB, with self-harm rates in affected youth up to 16%, and those who self-harm face a 37-fold increased risk of suicide within a year [23].

**Table 4:** Risk factors and intersectional vulnerabilities NSSIB: non-suicidal self-injury behavior, AD: atopic dermatitis, SGM: sexual and gender minority, LGBTQ+: lesbian, gay, bisexual, transgender, queer/questioning, BDD: body dysmorphic disorder, derm: dermatology or dermatologic, ↑: increase/higher, ↓: decrease/lower

Risk Factor	Condition(s)	Increased Risks	Notes	Key Studies
Childhood maltreatment	NSSIB, skin disease	5x ↑ NSSIB, ↑ psychiatric comorbidity	Trauma history is a strong predictor	Paller et al., 2024 [3]
Gender-affirming hormones	Acne (trans youth)	↑ Acne with testosterone, psychosocial distress	82% develop facial acne; consider for care	Ragmanauskaite et al., 2020 [17]
Race/colorism	All skin diseases	↑ discrimination, poor mental health	Colorism effect persists for women	Perreira et al., 2019 [5]
Disease visibility	All visible dermatoses	↑ stigma, bullying, anxiety/depression	Self/parent report > physician for impact	Paller et al., 2024 [3]; Stuhlmann, 2025 [12]
Social media use	Acne, AD, all youth	↑ loneliness, bullying, BDD risk	“Selfie phobia,” upward comparisons	Kelly et al., 2021 [14]; Hughes and Bewley, 2023 [22]
SGM/LGBTQ+ identity	Acne, all skin diseases	↑ depression, suicide, avoidance	Minority stress model	Gao et al., 2017 [16]; Yeung et al., 2019 [21]
Socioeconomic status	All	↑ stress, reduced care access	Financial barriers to derm care, self-medication	Sun et al., 2025 [1]; Vilar et al., 2015 [10]

Disparities in Indigenous, Latinx, and Migrant Youth

These mental health risks are even more pronounced in marginalized groups. Indigenous youth in the United States and Canada have the highest suicide rates among adolescents, a phenomenon linked to a complex interplay of discrimination, trauma, and lack of culturally competent care [6,19]. Latinx and migrant youth, particularly those with visible skin differences, are at amplified risk due to the intersection of skin disease-related stigma and ethnic/racial bullying, leading to increased anxiety, depression, and self-harm [5,6]. Colorism and discrimination within and across communities further increase the psychosocial burden for darker-skinned adolescents, especially girls [5]. Sexual and gender minority youth with acne and other visible skin conditions are another especially high-risk group, with national data showing over 35% of sexual and gender minority youth with acne experiencing suicidal ideation, nearly five times higher than their heterosexual peers with clear skin [17].

Underdiagnosis and Barriers to Mental Health Care

Despite the high prevalence of psychiatric comorbidities, mental health needs are frequently underrecognized and undertreated in adolescents with visible skin disease [1,3]. Stigma, shame, cultural taboos, and lack of mental health literacy lead many youth to conceal their psychological distress, particularly in Indigenous, migrant, and Latinx families where discussion of mental illness may be discouraged [6,12]. Socioeconomic barriers and a lack of insurance further limit access to care, with many youth relying on school-based or community resources, if available at all [9,21]. Dermatology clinics often miss the opportunity to screen for depression, anxiety, or suicidality, especially in busy or resource-limited settings [1,19]. Even when psychiatric needs are identified, referral pathways are often unclear or inaccessible, and youth from marginalized backgrounds face additional barriers due to language, mistrust, or prior discrimination within the healthcare system [3].

In summary, visible skin disease in adolescence is a major risk factor for depression, anxiety, self-harm, and suicidality, with the burden highest among Indigenous, Latinx, migrant, and gender-diverse youth. Routine mental health screening and culturally sensitive care pathways are urgently needed to address these disparities (Table 5).

**Table 5:** Prevalence of stigma, bullying, and mental health outcomes in marginalized youth with skin disease AD: atopic dermatitis, QoL: quality of life, PTSS: post-traumatic stress symptoms, PISS: psoriasis internalized stigma scale, peds: pediatrics, US: United States, “clinic”: clinic-based sample, “identity-based”: bullying/discrimination based on identity (e.g., race/ethnicity), “cyberbullying”: online/social media bullying, “comorbid”: co-occurring condition, “high visibility”: disease in visible body locations

Key References	Population/Condition(s)	Stigma Prevalence	Bullying/Teasing Prevalence	Major Psychosocial Outcomes	Notable Risk Modifiers	Key Findings/Quotes
Paller et al., 2024 [3]	1,671 children, chronic skin disease	73%	29%	Not specified	Disease severity, QoL, bullying	Stigma correlates with disease severity, QoL, and bullying
Cheng et al., 2023 [24]	US teens, AD	Not specified	33.2% (AD) vs. 19% (no AD); 9.1% vs 5.8% (cyberbullying)	Lower QoL, peer problems	AD, comorbid asthma	AD is associated with higher bullying risk, cyberbullying; bullying may mediate AD and poor QoL
Alvis et al., 2023 [25]	899 Black/Latino youth (clinic)	Not specified	34.1% identity-based	Higher depression, PTSS (girls > boys)	Identity-based bullying, gender	Identity-based bullying = higher depression, PTSS
Alpsoy et al., 2020 [26]	125 peds, 1,235 adults, psoriasis	High PISS: ~59-61	Not measured	Higher with stigma	-	Stigma is linked to lower QoL and mental health
Leszczynska et al., 2020 [27]	Pediatric acne, AD, psoriasis, etc.	Not specified	~50% in clinic sample	Depression, anxiety, physical symptoms, school avoidance	Visibility (face/arms), type of skin disease	“Bullying can have short- and long-lasting effects.”
Magin et al., 2008 [28]	Acne, eczema, psoriasis (ages 13–73)	Not specified	“Significant minority” (esp. adolescence)	Low self-esteem, embarrassment, anger	Adolescents, visible sites	“Teasing was universally negative and hurtful.”
Magin, 2013 [18]	Acne, AD, psoriasis, nevi, etc.	Not specified	Up to 60% (US AD patients), 44% (psoriasis)	Depression, self-harm, lower QoL	Boys (AD), high visibility	“There was a conspicuous lack of playful teasing.”
Kelly et al., 2021 [14]	Pediatric acne, AD, psoriasis	Not specified	44% psoriasis, 20% eczema	Suicidal ideation, depression, strained family	Age, gender (females more), disease severity	“Risk persists after adjusting for depressive symptoms.”

Coping and resilience: cultural and family factors

Family support is a powerful protective factor against the psychosocial burden of visible skin disease in adolescents, especially for those from marginalized backgrounds. When families respond to a child’s diagnosis with empathy, education, and empowerment, affected youth are more likely to develop adaptive coping skills and maintain self-esteem [2,9]. In contrast, over-accommodation, inconsistent discipline, or reinforcement of shame can undermine resilience and exacerbate emotional distress [2]. Traditional beliefs and cultural pride also shape the lived experience of visible difference. In some communities, keloids and scars may be imbued with cultural significance or viewed as markers of identity, reducing their psychosocial impact [9]. For many Indigenous, Latinx, and migrant families, storytelling, ceremony, and collective memory offer frameworks for resilience, affirming the value of the individual and reframing visible difference as a source of strength rather than shame [6]. Positive cultural identity, connection to ancestral practices, and intergenerational wisdom can buffer the negative effects of discrimination and internalized stigma [5,6]. Culturally rooted pride and collective advocacy have been shown to mitigate the harmful effects of bullying and foster a sense of belonging, especially in youth who face both skin disease-related stigma and ethnic/racial discrimination [6].

Peer Networks, Storytelling, and Mentorship

Peer support networks, whether through support groups, social media, or school-based programs, are vital for reducing isolation and building resilience among adolescents with visible skin conditions [2,3]. Peer mentorship and storytelling create safe spaces for youth to share experiences, process stigma, and develop healthy coping strategies. Group interventions such as summer camps for children with chronic skin disease have been shown to improve adaptation, reduce feelings of shame, and help youth separate their core identity from their condition [2]. Mentorship by older youth or adults who have navigated similar challenges can model adaptive coping, reinforce self-worth, and foster long-term resilience [12]. Family-centered and community-based interventions are especially important for marginalized youth, who may face barriers to formal mental health care. Empowering families and leveraging community strengths can transform the experience of visible difference from one of isolation to one of connection, meaning, and resilience [6,9].

Interventions and emerging therapies

A growing body of research highlights the importance of comprehensive, multidisciplinary interventions to address the mental health and social challenges faced by adolescents with visible skin diseases. Effective strategies now extend beyond traditional dermatologic care, incorporating evidence-based psychological therapies, peer and family support, school-based anti-bullying programs, and digital health resources. Innovative approaches, including trauma-informed care, telepsychiatry, and culturally tailored interventions, are increasingly recognized for their potential to improve resilience and quality of life in marginalized youth. This section reviews the range of emerging therapies and integrated care models that have been shown to reduce stigma, support coping, and promote positive psychosocial outcomes in this vulnerable population.

Psychological Interventions

Effective psychosocial care for adolescents with visible skin disease increasingly includes evidence-based psychological interventions. Cognitive-behavioral therapy has shown efficacy for managing anxiety, depression, and body image disturbance in affected youth [9,23]. Group therapy and support groups foster peer connection, normalize shared experiences, and reduce isolation [2,3].

Telepsychiatry offers a promising avenue to increase access, particularly for minoritized and rural youth facing barriers to in-person care. Digital mental health resources, such as virtual support groups and self-help modules, can supplement traditional therapy, offering privacy and flexibility for youth reluctant to seek face-to-face help [9]. School-based programs that incorporate routine psychosocial screening and on-site counseling services help identify at-risk youth early and facilitate timely referral to mental health professionals [3,7]. Early identification and intervention are critical, given the high rates of underdiagnosis and stigma-related barriers in this population [1].

School and Community-Based Anti-bullying Programs

School and community-based anti-bullying programs are essential for reducing the risk of victimization, isolation, and secondary trauma among adolescents with visible skin disease [6,7]. Programs that directly address stigma, discrimination, and cultural diversity have demonstrated stronger impacts than those that focus only on general bullying prevention. Key features of successful interventions include multicultural education, peer-led campaigns, restorative justice practices, and active involvement from families and communities [6]. Interventions that empower students to share their stories and build empathy within the school community help counteract negative stereotypes and foster a culture of inclusion.

Dermatology-Mental Health Collaborations and Advocacy

Integration of dermatology and mental health care is increasingly recognized as a gold standard in the management of psychodermatologic conditions [1,23]. Collaborative models, such as psychocutaneous clinics, liaison services, and joint case conferences, facilitate bidirectional referrals and comprehensive, trauma-informed care [1,9]. Advocacy efforts at the institutional and policy levels are needed to expand training in psychodermatology, increase funding for integrated clinics, and address health disparities in marginalized youth. Dermatologists, mental health professionals, and community advocates can collaborate to raise awareness, develop culturally competent resources, and ensure that psychosocial needs are addressed in conjunction with medical care [1,21].

Recommendations and future directions

Improving psychosocial outcomes for marginalized adolescents with visible skin diseases will require a sustained, multi-level approach across clinical, educational, and community settings. Key recommendations include routine psychosocial screening in dermatology and pediatric clinics, stronger collaboration among healthcare providers, schools, and families, and the implementation of culturally sensitive, trauma-informed care models. Addressing critical research gaps, such as the need for validated stigma measures and long-term intervention studies, will be essential for guiding future policy and practice. This section outlines actionable steps and research priorities aimed at reducing disparities, enhancing support systems, and empowering youth to thrive despite the challenges of visible skin disease.

Integrated Clinical and School-Based Collaboration

Improving outcomes for marginalized adolescents with visible skin disease requires active collaboration between dermatologists, pediatricians, school personnel, and mental health professionals (Table 6). Routine psychosocial screening for depression, anxiety, and stigma should be standard in dermatology and pediatric clinics, using validated tools such as the PROMIS PPS-Skin or Children’s Dermatology Life Quality Index (CDLQI) [3,11]. School counselors, nurses, and teachers must be educated to recognize early warning signs of distress or bullying and facilitate referrals to appropriate resources [6,7].

**Table 6:** Key roles in integrated care for marginalized youth with visible skin disease PROMIS PPS-Skin: Patient-Reported Outcomes Measurement Information System PROMIS Pediatric Stigma, CDLQI: Children’s Dermatology Life Quality Index, “high-barrier populations”: communities facing significant healthcare or resource obstacles, “trauma-informed care”: approaches that recognize and address the impact of trauma

Stakeholder	Key Responsibilities
Dermatologists and pediatricians	Routine psychosocial screening (PROMIS PPS-Skin, CDLQI); ask about peer relationships and school experiences; formal referral networks with school-based professionals; community outreach in high-barrier populations
School personnel	Recognize early signs of distress/bullying; facilitate referral to resources; support anti-bullying programs; participate in mental health promotion
Mental health providers	Receive rapid referrals; collaborate with clinical and school teams; provide trauma-informed care
Community leaders	Promote cultural pride, resilience, and support; bridge traditional and biomedical approaches

Policy and Education Initiatives

A striking gap remains in psychodermatology education for healthcare professionals and school staff [1]. Curricula at the medical and graduate education level must include: basic psychodermatology principles; culturally competent, trauma-informed care; and strategies for stigma reduction and mental health promotion in youth with visible differences.

Schools and health systems should implement and enforce anti-bullying and anti-discrimination policies, with special attention to intersectional identities [6]. Policy-makers and professional organizations should advocate for expanded funding and research in the field of psychodermatology, with a focus on marginalized youth.

Research Gaps and Future Needs

Critical gaps persist in the research on intersectional stigma, internalized shame, and the longitudinal outcomes of psychosocial interventions for youth with visible skin disease (Table 7) [3,12].

**Table 7:** Research gaps and future needs QoL: quality of life, “intersectional stigma”: stigma arising from overlapping marginalized identities, “internalized shame”: self-directed negative feelings related to social stigma, “digital and integrative interventions”: technology-based or complementary/holistic therapies, “high-risk/resource-limited populations”: groups facing elevated vulnerability and/or barriers to care

Gap/Need	Action/Research Priority
Intersectional stigma and internalized shame	Develop/validate adolescent- and culture-specific screening tools
Long-term outcomes of interventions	Study the impact of early intervention on mental health, achievement, and QoL
Cultural/community support	Examine the roles of storytelling, art, and ceremony in resilience
Digital and integrative interventions	Test digital and integrative therapies in high-risk/resource-limited populations

Practical Tips for Providers and Educators

Meeting the psychosocial needs of marginalized youth with visible skin disease will require coordinated, trauma-informed care across clinical, school, and community settings. Closing the gaps in provider education, research, and policy is not only a matter of equity but an essential step in promoting health, dignity, and belonging for all adolescents (Table 8) [1,3,12].

**Table 8:** Practical tips for providers and educators PROMIS PPS-Skin: Patient-Reported Outcomes Measurement Information System PROMIS Pediatric Stigma, CDLQI: Children’s Dermatology Life Quality Index, CBIS: Child Body Image Scale, “anticipatory guidance”: proactive education about expected challenges, “shared decision-making”: collaborative care planning between providers, patients, and families

Practice	Details/Examples
Normalize discussion	Routinely ask about self-image, peer relationships, and bullying
Use validated tools	PROMIS PPS-Skin, CDLQI, CBIS as part of routine care
Early referral	Clear, rapid protocols for referring at-risk youth to mental health services
Culturally tailored care	Involve families, cultural liaisons, and community leaders for holistic, respectful care
Empower and educate	Provide anticipatory guidance, support shared decision-making, and build resilience

## Conclusions

The experiences of adolescents living with visible skin diseases, especially those from Indigenous, Latinx, migrant, and other marginalized communities, underscore the urgent need for a more holistic and culturally attuned model of care. The psychosocial impact of skin conditions extends far beyond physical symptoms, affecting identity formation, self-esteem, academic achievement, and mental health during some of the most formative years of life. For many, these challenges are compounded by the intersecting pressures of systemic discrimination, cultural stigma, socioeconomic barriers, and historical trauma. Moving forward, dermatologists, pediatricians, mental health professionals, schools, and families must work collaboratively to create environments where all youth feel safe, understood, and supported. Trauma-informed, culturally competent care should become the standard, incorporating routine screening for psychological distress, open conversations about stigma, and family-centered approaches that honor traditional beliefs and strengths. School- and community-based interventions, peer mentorship, and creative therapies, such as art and storytelling, can be powerful tools in building resilience and fostering a sense of belonging. Moreover, it is imperative to address the research and policy gaps that limit equitable access to comprehensive care. This includes investing in longitudinal studies that track psychosocial outcomes, developing validated screening tools for diverse populations, and advocating for insurance and educational reforms that recognize the unique burdens faced by minoritized youth with skin disease. Ultimately, fostering holistic well-being for these adolescents requires a commitment to viewing the patient not only as an individual with a skin condition but as a whole person shaped by culture, community, and lived experience. By embracing this integrative approach, the medical and educational communities can help dismantle barriers, reduce stigma, and empower young people to thrive despite the visible differences they may carry.
